# Bulimia nervosa in times of the COVID‐19 pandemic—Results from an online survey of former inpatients

**DOI:** 10.1002/erv.2773

**Published:** 2020-08-07

**Authors:** Sandra Schlegl, Adrian Meule, Matthias Favreau, Ulrich Voderholzer

**Affiliations:** ^1^ Department of Psychiatry and Psychotherapy University Hospital (LMU) Munich Germany; ^2^ Schoen Clinic Roseneck Prien am Chiemsee Germany; ^3^ Department of Psychiatry and Psychotherapy University Hospital Freiburg Germany

**Keywords:** bulimia nervosa, coping strategies, COVID‐19 pandemic, health care utilisation, relapse, symptom deterioration

## Abstract

**Objective:**

The COVID‐19 pandemic might pose special challenges to patients with eating disorders (EDs) by interfering with daily routines. The aim of this study was to investigate the impact of the current pandemic on patients with bulimia nervosa (BN).

**Methods:**

Fifty‐five former inpatients with BN completed an online survey on psychological consequences of the COVID‐19 pandemic as well as on changes in health care utilisation and on the use and helpfulness of different coping strategies.

**Results:**

Almost half of patients (49%) reported a deterioration of their ED symptomatology and 62% reported a reduced quality of life. The frequency of binge eating increased in 47% of patients and self‐induced vomiting in 36%. Forty‐six percent of patients stated a noticeable impairment of psychotherapy. Face‐to‐face psychotherapy decreased by 56% but videoconferencing therapy was only used by 22% of patients. Enjoyable activities, virtual social contacts with friends and mild physical activities were rated as the most helpful coping strategies among those most used.

**Discussion:**

Approximately one half to two‐thirds of former inpatients with BN experienced a negative impact of the crisis on their ED symptomatology and quality of life. In challenging times when face‐to‐face therapy options are restricted, e‐health treatments such as videoconferencing therapy should be considered to ensure continuity of care.

**Highlights**

- During the COVID‐19 pandemic one‐third to half of patients with bulimia nervosa (BN) reported an increase in their bulimic symptomatology.
- Furthermore, depressive symptoms, general psychopathology, quality of life and therapy in patients with BN were negatively affected.
- The utilisation rate of e‐mental health interventions during the crisis was rather low.
- The most useful coping strategies (among those most used) were enjoyable activities, virtual social contacts with friends and mild physical activities.

## 1 INTRODUCTION

Changes in our everyday life associated with the COVID‐19 pandemic pose great challenges to all of us. There is a high awareness that individuals with mental disorders are affected significantly more by external stressors than resilient persons. One reason for this is that persons with mental disorders in general—and those with eating disorders (EDs) in particular—tend to use more dysfunctional emotion regulation or coping strategies than individuals without mental disorders (Aldao, Nolen‐Hoeksema, & Schweizer, 2010). However, there are few data on how patients with EDs cope with the current global crisis.

Recently, an international online survey examined the impact of the COVID‐19 pandemic on eating behaviour and physical activity in the general population. It was found that physical activity decreased and eating behaviour (type of food consumption, eating out of control, snacks between meals, number of main meals) was more unhealthy during confinement than before (Ammar et al., 2020). Other studies have documented an increase in anxiety, depression, and loneliness (Killgore, Cloonan, Taylor, & Dailey, 2020; Li et al., 2020).

In addition to the effects of the pandemic on the general population's well‐being, it has been widely recognised that the pandemic may potentially facilitate symptom deterioration and relapse in patients with EDs in particular (Fernandez‐Aranda et al., 2020; Todisco & Donini, 2020; Touyz, Lacey, & Hay, 2020; Weissman, Bauer, & Thomas, 2020).

Indeed, preliminary data suggest that many patients with EDs show a worsening of their overall ED symptomatology. However, estimates vary substantially between studies (between 38% and 87%) and between specific ED‐related behaviours (Branley‐Bell & Talbot, 2020; Fernandez‐Aranda et al., 2020; Phillipou et al., 2020; Schlegl, Maier, Meule, & Voderholzer, in revision; Termorshuizen et al., 2020). Moreover, most of these studies have examined heterogeneous samples or focused on anorexia nervosa (AN). Therefore, a gap exists in the extant literature regarding the effects of the pandemic in individuals with bulimia nervosa (BN).

So, the aim of this brief report is to report on the psychological consequences of the COVID‐19 pandemic as well as changes in health care utilisation and the use and usefulness of possible strategies to cope with these times in patients with BN.

## 2 METHODS

### 2.1 Study participants

The study was approved by the institutional review board of the Medical Faculty of the Ludwig Maximilian University Munich, Germany. Two hundred and three former inpatients with BN who had received treatment in the Schoen Clinic Roseneck (Prien am Chiemsee, Germany) and who were discharged in 2018 and 2019 were contacted by email and invited to complete an anonymous online survey (lasting approximately 15 min) via www.unipark.com. Inclusion criteria were (a) having a primary diagnosis of BN (ICD‐10: F50.2 or F50.3) at admission, (b) being female, and (c) being at least 13 years old. Germany has been in lockdown approximately from end of March to end of April 2020 (depending on the region), and the survey was conducted in the first week of May 2020. One reminder was sent 1 week later.

### 2.2 Instruments

We used a self‐developed questionnaire equivalent to the survey of patients with AN (Schlegl et al., in revision) to assess psychological consequences of the COVID‐19 pandemic. It was divided into seven parts: (a) Socio‐demographic and other information such as age, sex, current self‐reported height and weight, occupational situation during the COVID‐19 pandemic, and contact history with severe acute respiratory syndrome coronavirus 2 (SARS‐CoV‐2), that is, current or previous own confirmed infection or infection of others (yes/no answers); (b) Overall impact of the COVID‐19 pandemic on ED symptoms and general well‐being, that is, deterioration of symptomatology and quality of life, adverse effects on therapy, incidence of new symptoms (5‐point scale with 1 = *strongly agree* to 5 = *strongly disagree*); (c) Worries, for example, regarding infections, relapses, food insecurity, finances, and job (5‐point scale with 1 = *extremely worried* to 5 = *not at all worried*); (d) General psychopathology and interpersonal conflicts (5‐point scale with 1 = *significantly worsened* to 5 = *significantly improved*); (e) Specific ED symptoms and behaviours (5‐point scale from 1 = *significantly worsened/much more* to 5 = *significantly improved/much less*); (f) Health care utilisation before and during the COVID‐19 pandemic (yes/no answers); (g) Use and helpfulness of strategies as suggested by Fernandez‐Aranda et al. (2020) (combination of yes/no answers and a 5‐point scale with 1 = *not helpful at all* to 5 = *very helpful*).

### 2.3 Statistical analyses

Responses to all questions are reported descriptively (relative frequencies, means). For questions that had a 5‐point scale response format, note that percentages reported in the main text in the results section represent the combination of the two categories that indicate endorsement to facilitate readability. That is, percentages of endorsement of each statement refer to the combined percentages of the categories *agree/strongly agree*, *moderately worried/extremely worried*, *somewhat worsened/significantly worsened*, and *more /much more*. All analyses were conducted with IBM SPSS Statistics version 24.

## 3 RESULTS

### 3.1 Socio‐demographic and other information

In total, 91 of the 203 former inpatients with BN who were contacted accessed the survey. Of these 91 patients, 15 patients only viewed the first page of the survey, 2 patients did not agree to informed consent and 74 patients gave electronic informed consent. The final sample comprised the 55 patients (27.1% of the total sample) who completed the online survey. Mean age was 24.42 years (*SD* = 6.36, range: 17–46, *n* = 2 minors). Mean body mass index was 23.62 kg/m^2^ (*SD* = 4.58). During the COVID‐19 pandemic, the occupational situation of patients was homeschooling (20.0%), university online classes (20.0%), working from home (10.9%), working at workplace (21.8%), reduced working hours (3.6%), job loss due to the COVID‐19 pandemic (3.6%) and others (20.0%). One patient was infected with SARS‐CoV‐2, two reported an infection in the household and three reported an infection among related parties.

### 3.2 Impact of the COVID‐19 pandemic on patients with BN

**FIGURE 1:**
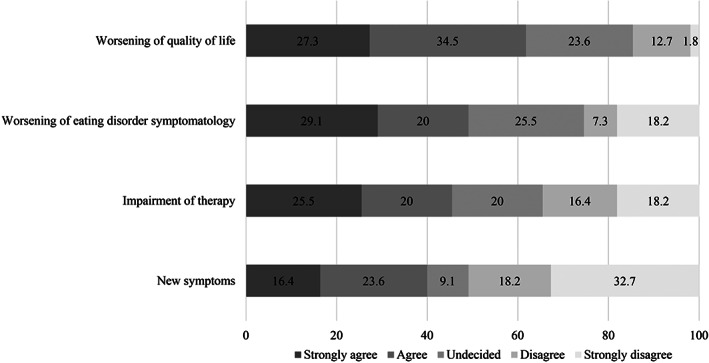
Percentages of the overall impact of the COVID‐19 pandemic on patients with bulimia nervosa

**FIGURE 2:**
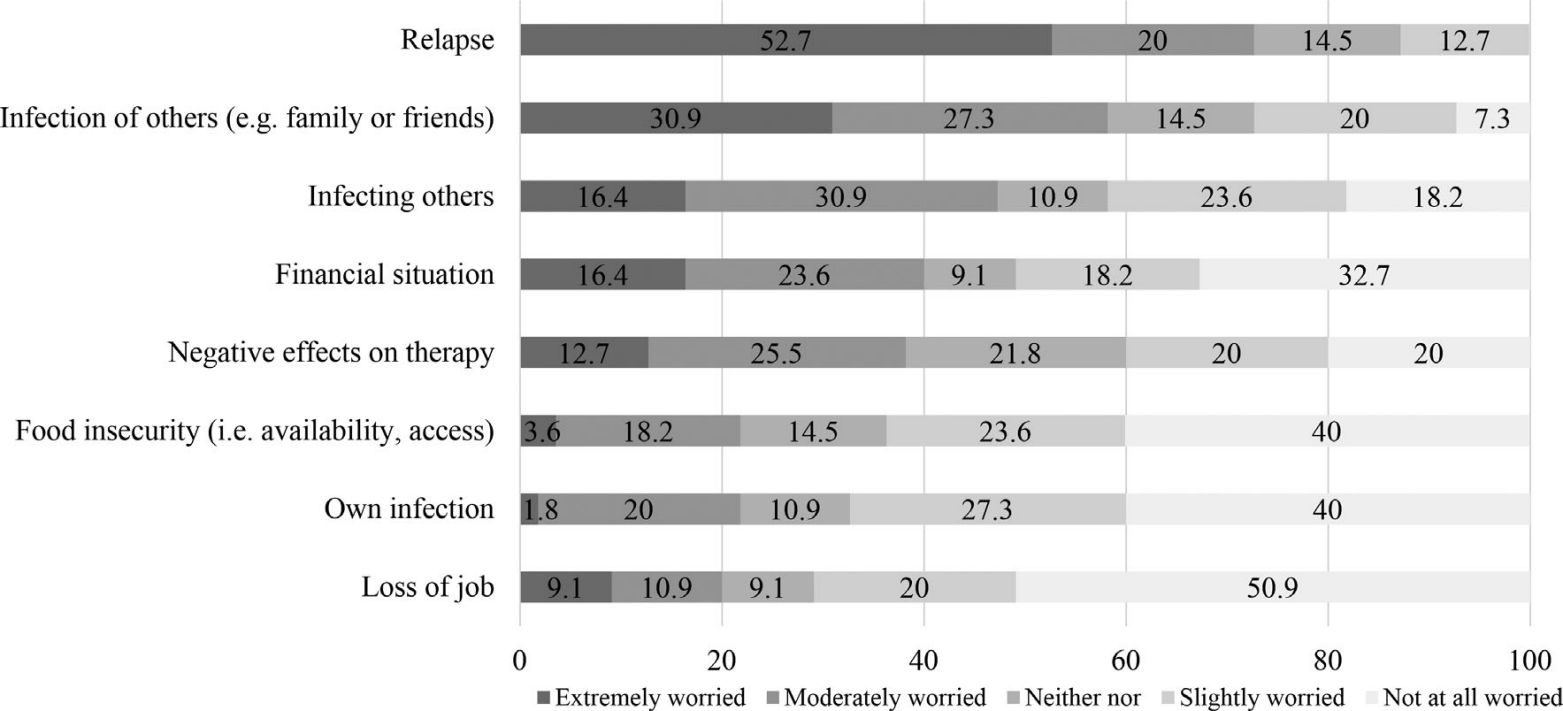
Percentages on how much patients with bulimia nervosa were worried during the COVID‐19 pandemic

**FIGURE 3:**
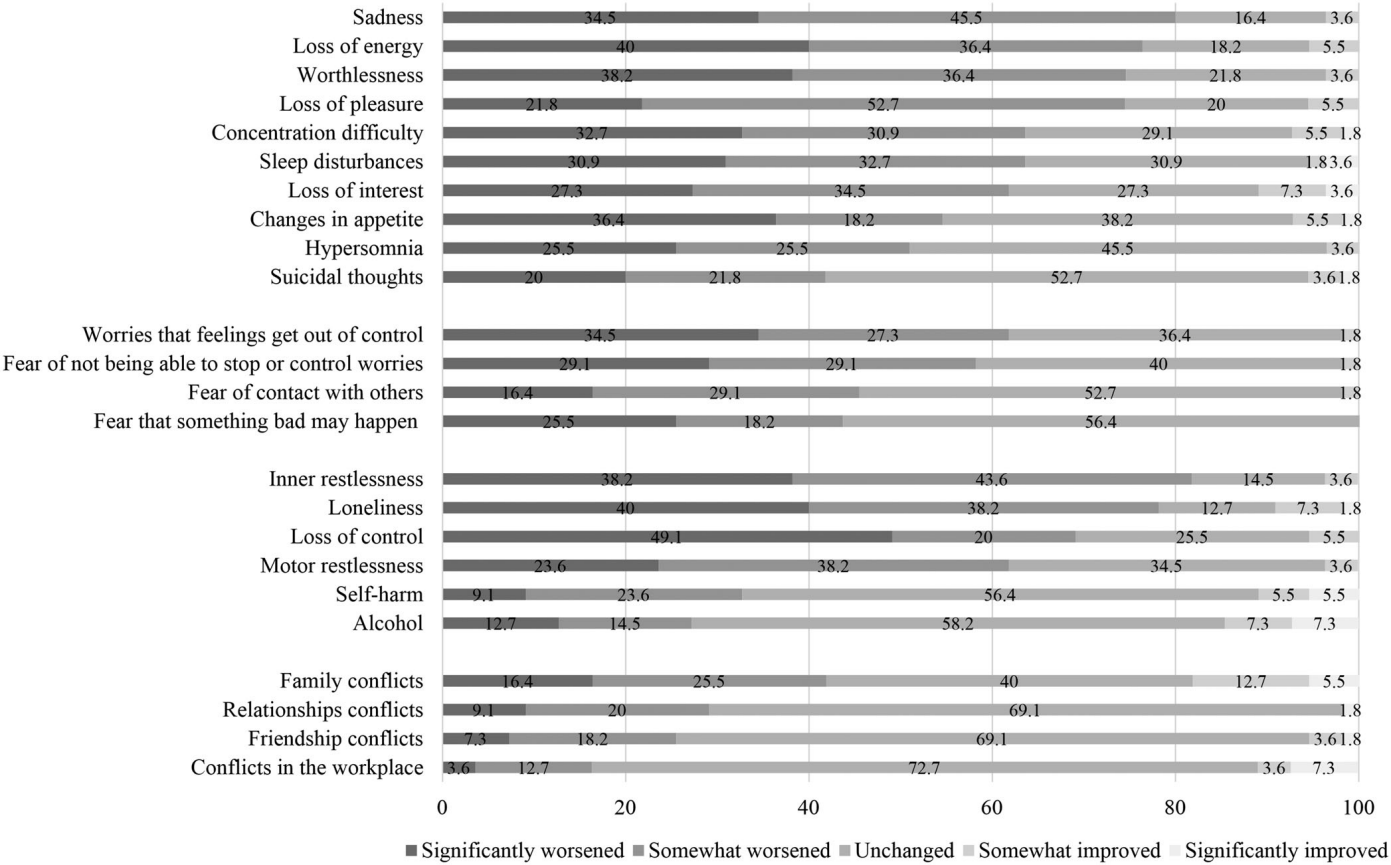
Percentages on how general psychopathology and interpersonal conflicts in patients with bulimia nervosa changed during the COVID‐19 pandemic

**FIGURE 4:**
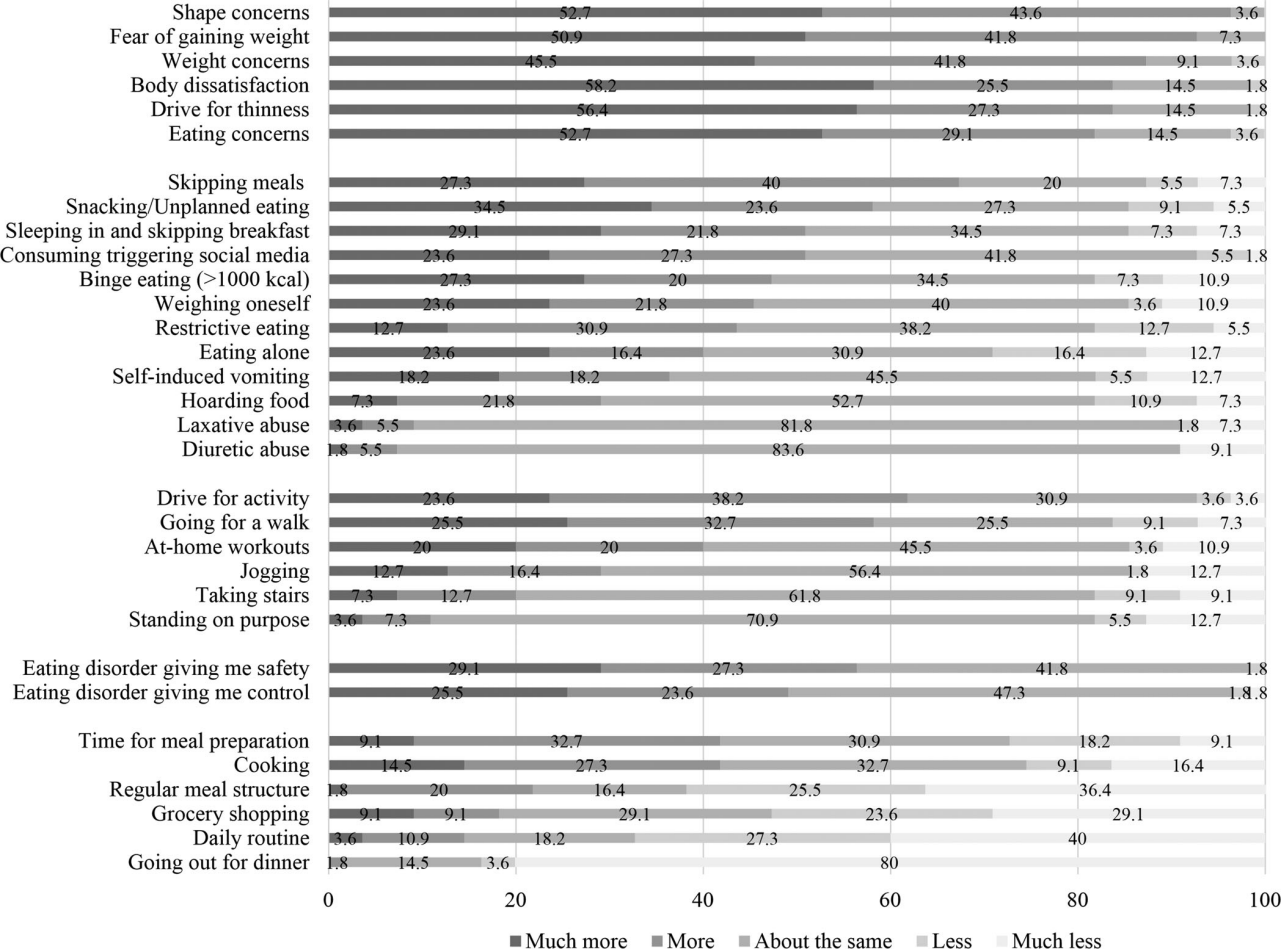
Percentages on how eating disorder symptoms and behaviours changed in patients with bulimia nervosa during the COVID‐19 pandemic

The overall impact of the COVID‐19 pandemic on ED symptoms and general well‐being, worries, the impact on general psychopathology, interpersonal conflicts as well as specific ED symptoms and behaviours are shown in Figures 1, 2, 3, 4.

Almost half of patients with BN reported a worsening of their ED symptomatology (49.1%) and 61.8% of their quality of life. Furthermore, 45.5% expressed a significant impairment of current psychotherapy and 40.0% reported that they developed new symptoms.

Sadness, loss of energy, inner restlessness, and loneliness were the most pronounced depressive and general psychopathology symptoms (over 75%). Furthermore, shape, weight, and eating concerns as well as fear of gaining weight, body dissatisfaction, and drive for thinness increased in most patients (over 80%). A higher drive for activity was prevalent in 61.8% of patients and 61.9% reported a less or much less‐regular meal structure. Binge eating increased in 47.3% of patients, self‐inducing vomiting in 36.4%, laxative use in 9.1% and diuretic abuse in 7.3% of patients. On the contrary, a quarter of patients reported more or much more time for meal preparation and more or much more cooking.

### 3.3 Health care utilisation

More than 80% of patients with BN received face‐to‐face therapy before the COVID‐19 pandemic (81.8%) compared to 36.4% during the pandemic (i.e., a decrease by 55.5%). Use of videoconference‐based therapy increased from 3.6% to 21.8% and use of telephone contacts from 18.2% to 38.2%, whereas the use of additional online interventions decreased from 3.6% to 0%.

### 3.4 Use and helpfulness of strategies

**FIGURE 5:**
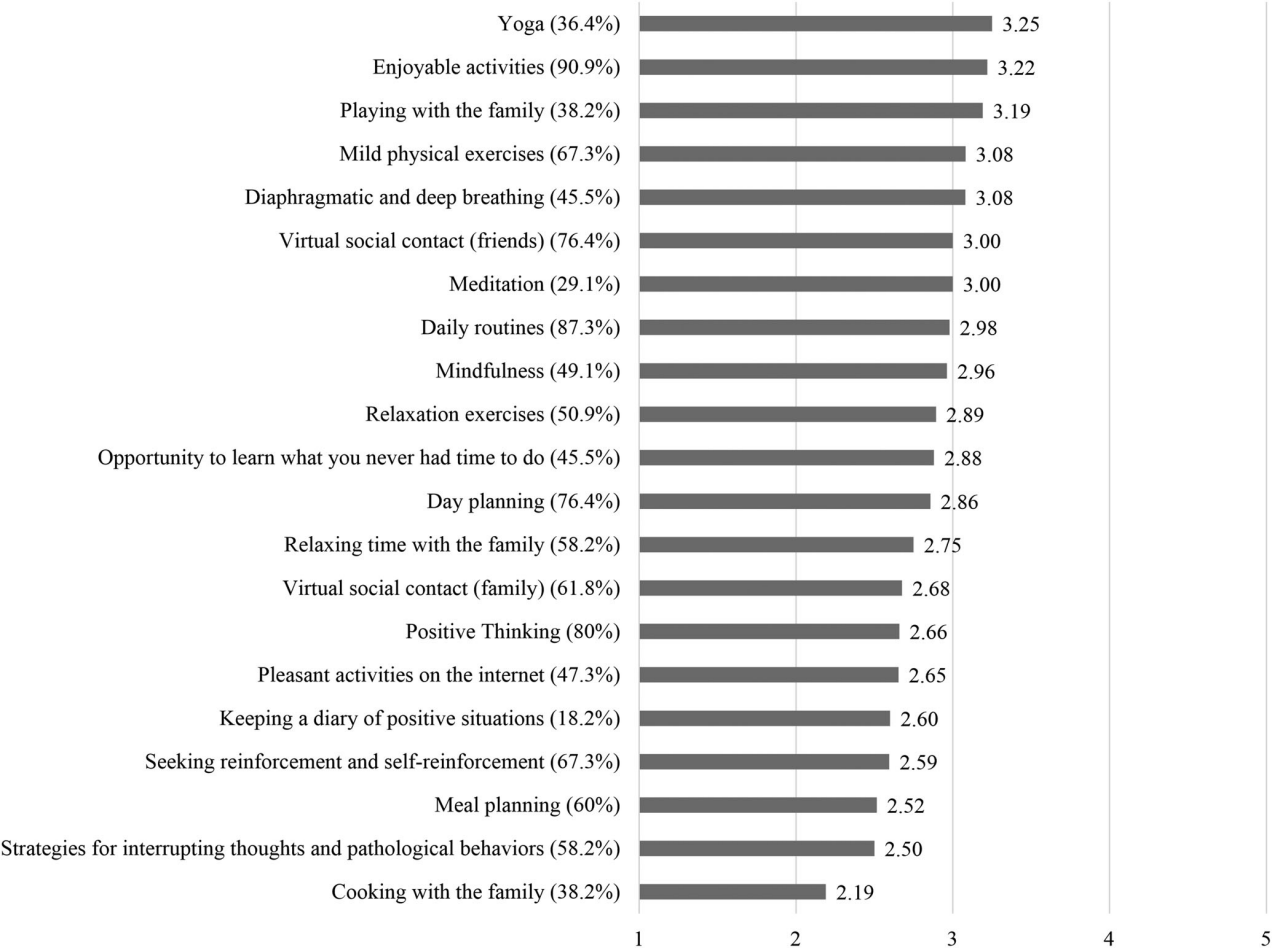
Mean responses to questions on helpfulness of strategies. Higher scores represent higher helpfulness ratings. The values in parentheses reflect the percentages of patients that used the strategies. 1 = not helpful at all to 5 = very helpful

Enjoyable activities, virtual social contact with friends and mild physical exercises were the strategies rated as most helpful among those most used. Figure 5 shows mean responses to questions on helpfulness of different strategies for patients with BN during the COVID‐19 pandemic.

## 4 DISCUSSION

### 4.1 Summary of results

More than half of former inpatients with BN experienced a worsening of their ED symptomatology as well as of their quality of life during the crisis. Furthermore, depressive and general psychopathology symptoms increased in up to 80% of patients. Binge eating increased in nearly half of the patients, self‐induced vomiting in one third, laxative use and diuretic abuse in almost 10%. A higher drive for activity was prevalent in more than 60% of patients. More than 60% of patients also reported problems with maintaining their established daily routine and regular meal structure. Furthermore, more than half of patients could not continue their face‐to‐face therapy. Yet, only 20% of patients used alternative treatment modalities such as videoconference‐based therapy.

### 4.2 Comparison with findings of other studies

Compared to the impact that the COVID‐19 pandemic had on former inpatients with AN (Schlegl et al., in revision)—with 42% reporting a worsening of their ED symptomatology and 52% a worsening of their quality of life—the impact of the crisis on ED symptoms seems even stronger in patients with BN. Furthermore, almost twice as many patients with BN than those with AN reported a significant impairment of therapy during the crisis and the development of new symptoms.

Compared to international studies, our value for a worsening of overall symptomatology (49.1%) lies in the middle of the range (38.0%–86.7%) (Branley‐Bell & Talbot, 2020; Fernandez‐Aranda et al., 2020). However, we found an almost twofold increase in binge eating and urges to binge compared to the U.S./Netherlands sample sub‐group of patients with BN and Binge eating disorder (47.3% vs. 15%–30%) (Termorshuizen et al., 2020). This might be at least partially attributable to our survey of a more severely ill sample (former inpatients with a still high health care utilisation rate) compared to the sample of the other study where only half of patients were in treatment at the beginning of the COVID‐19 pandemic.

### 4.3 Effects on therapy and use of e‐health treatments

The utilisation rate of videoconference‐based therapy of only 20% in our German sample is much lower than in the United States and Netherlands where 42%–45% of patients transitioned to online/tele‐health care (Termorshuizen et al., 2020). Therefore, feasibility and/or acceptance of e‐mental health treatments in patients with BN do not seem very high, at least in Germany.

Several suggestions exist on how to deliver evidence‐based treatments such as cognitive‐behavioural therapy or family‐based treatment remotely in times of contact restrictions (Matheson, Bohon, & Lock, 2020; Murphy, Calugi, Cooper, & Dalle Grave, 2020; Touyz et al., 2020; Waller et al., 2020). Furthermore, there is one randomized controlled trial (RCT) showing a comparable efficacy of telemedicine and face‐to‐face treatment in patients with BN (Mitchell et al., 2008). Additionally, online interventions such as “Overcoming Bulimia Online”—which has been evaluated in an RCT (Sánchez‐Ortiz et al., 2011)—might in principle be an option, but unfortunately are not available in German. Also, the smartphone App “Recovery Record” might be a helpful tool, either as self‐help or as therapist‐guided intervention (Tregarthen et al., 2015). One of the main “Recovery Record” elements is self‐monitoring. Self‐monitoring of meals might help to keep a regular, adequate meal structure, which in turn might help to prevent binge eating and subsequent compensatory behaviour. As “Recovery Record” includes these protocols and inquiries about urges as well as disordered behaviour, both patient and therapist can detect early warning signs of relapse more easily, which allows for timely intervention.

### 4.4 Strengths and limitations of the current study

Strengths of our study were that we investigated a sample with a confirmed clinical diagnosis of BN. Furthermore, we performed a comprehensive query of data regarding a wide range of potential detrimental effects of the COVID‐19 pandemic on patients with BN, but we also focused on potential helpful coping strategies. However, there are also some limitations that should be considered: The completion rate of 27% was relatively low. It is possible that those who were not considerably affected by the COVID‐19 pandemic were less likely to respond. However, in a sensitivity analysis (not reported), we found no differences in answers between those who responded to the first invitation or the reminder. Furthermore, increasing response rates do not necessarily reduce such a bias (Dillman, Eltinge, Groves, & Little, 2002). Furthermore, as our data are cross‐sectional, we do not know if these shown effects are only temporary or will have a long‐lasting negative impact.

### 4.5 Future research

Follow‐up surveys will be important to shed more light on the last raised point. Additionally, further RCTs comparing face‐to‐face therapies and e‐mental‐health interventions and/or blended treatments and/or evaluating patients who switched from face‐to‐face to e‐mental‐health interventions regarding outcome and therapeutic alliance might be highly warranted to ascertain if and to what extent evidence‐based therapy can also be provided remotely. Finally, investigating barriers and facilitating factors for remote therapy seems worth researching since it might be a promising solution to support patients best during such crises.

### 4.6 Conclusion

The current pandemic had and likely continues to have detrimental effects on symptomatology and treatment of patients with BN. However, we should not forget that the COVID‐19 pandemic might also be a chance for patients with BN to learn key life skills such as coping with life in general, with emotions and stress, to change their way of thinking and to set new goals. Being able to offer them evidence‐based alternative therapeutic treatment strategies should be a goal for future.

## CONFLICT OF INTEREST

We have no conflicts of interest to declare.

## Data Availability

The data that support the findings of this study are available from the corresponding author upon reasonable request.
